# Guidelines for confidentiality and cancer registration

**DOI:** 10.1038/sj.bjc.6602618

**Published:** 2005-05-10

**Authors:** H Storm, D H Brewster, M P Coleman, D Deapen, A Oshima, T Threlfall, E Démaret

**Affiliations:** 1 Danish Cancer Society, Copenhagen, Denmark; 2 Scottish Cancer Registry, Edinburgh, Scotland, UK; 3 Non-Communicable Disease Epidemiology Unit, London School of Hygiene and Tropical Medicine, London, UK; 4 Los Angeles Cancer Surveillance Program, Los Angeles, USA; 5 Osaka Cancer Registry, Osaka, Japan; 6 Western Australian Cancer Registry, Perth, Australia; 7 Descriptive Epidemiology Group, International Agency for Research on Cancer, Lyon, France

**Sir**,

Legislation and professional guidance on confidentiality in medical research has increased significantly in the past 10 years (Stiller, 1993; Working Group to the Royal College of Physicians Committee on Ethical Issues in Medicine, 1994; European Parliament, 1995; The Caldicott Committee, 1997; Department of Health, 1999; General Medical Council, 2000; Medical Research Council, 2000; Coker and McKee, 2001; Confidentiality and Security Advisory Group for Scotland, 2002; Council for International Organizations of Medical Sciences, 2002; Information Commissioner, 2002).

Numerous reports have been issued by national and international bodies (Lowrance, 1997; National Health and Medical Research Council, 1999; Canadian Institutes of Health Research, 2001; Organisation for Economic Co-operation and Development, 2001; Council for International Organizations of Medical Sciences, 2002; Lowrance, 2002; Nuffield Council on Bioethics, 2002; World Medical Association, 2002a, 2002b; Medical Research Council, 2003; National Institutes of Health, 2004).

There is very wide debate over the appropriate balance to be struck between increasing demands for personal autonomy, on the one hand, and, on the other, the need for society to learn from the experience of individual patients, in order to understand how best to control disease – this is also in the interests of individuals. The debate has often focused on the confidentiality of individual health data and the need for informed consent before such data can be used in research (Vandenbroucke, 1992; Vanchieri, 1993; Strobl *et al*, 2000; Anderson, 2001; Bastian, 2001; Doll, 2001; Doll and Peto, 2001; Cassell and Young, 2002; Greenberg, 2002; Kulynych and Korn, 2002a, 2002b; Verity and Nicoll, 2002; Coleman *et al*, 2003; De Vet *et al*, 2003; Ingelfinger and Drazen, 2004; Peto *et al*, 2004; Tu *et al*, 2004; Robling *et al*, 2005).

The International Association of Cancer Registries (IACR) published guidance on confidentiality for cancer registries in the *British Journal of Cancer* in 1992 (Coleman *et al*, 1992). Some national and regional cancer registry associations incorporated the IACR guidance in their own guidelines. At the IACR scientific meeting in Tampere, Finland, in 2002, it was decided to update the guidance. A review seemed appropriate after 10 years. European Union (EU) legislation on the protection of personal data had come into force in all member states during this period, and the EU Directive (European Parliament, 1995) has served as a model for national legislation in many countries outside Europe. Rapid developments in web-based communication also motivated revision of the guidance, with a view to appropriate use of this technology, with the attendant risks of breach of confidentiality. The guidance was revised by a small group, endorsed by the IACR Board in 2004, and made available at www.iacr.com.fr/confidentiality2004.pdf.

The main changes from the previous version are:

- a clear description of the principles of confidentiality, as they relate to identifiable data and the registration of cancer;
- an update of measures to protect data confidentiality;
- guidance on security for both traditional paper-based systems and modern electronically based data systems; and
- expanded recommendations designed to ensure confidentiality in data releases for research, including cross-border transfers.

The updated IACR guidance on confidentiality in the cancer registry should help the cancer research community continue to provide useful information on the causes, treatment and outcome of cancer in the entire population, while maintaining the highest ethical standards in confidential data collection and research.
